# Effect of Extracellular Matrix Derived from Porcine Tissue on Stemness of Porcine Spermatogonial Stem Cells

**DOI:** 10.3390/ijms26209937

**Published:** 2025-10-13

**Authors:** Donghyeon Kim, Min-Gi Han, Yoseop Jeon, Hyoyoung Maeng, Youngseok Choi, Kwonho Hong, Jeong Tae Do, Hyuk Song

**Affiliations:** 1 School of Advanced Biotechnology, Konkuk University, Seoul 05029, Republic of Korea; kdh2987@konkuk.ac.kr (D.K.); hmg82@konkuk.ac.kr (M.-G.H.); jeonjoseph@konkuk.ac.kr (Y.J.); mang0724@konkuk.ac.kr (H.M.); choiys3969@konkuk.ac.kr (Y.C.); hongk@konkuk.ac.kr (K.H.); dojt@konkuk.ac.kr (J.T.D.); 2 The Institute of Advanced Regenerative Sciences, Konkuk University, Seoul 05029, Republic of Korea

**Keywords:** porcine spermatogonial stem cells (pSSCs), extracellular matrix (ECM), pECM coating, Sertoli cell, stemness maintenance

## Abstract

The extracellular matrix (ECM) supports spermatogonial stem cell (SSC) function by mimicking biochemical and structural features of the native niche. However, optimal feeder systems and ECM materials remain key limitations in porcine SSC (pSSC) cultures. We developed a porcine-derived ECM (pECM) from porcine feet and evaluated its effectiveness in supporting pSSC maintenance and proliferation under feeder-dependent conditions. We examined protein molecular weight distribution and pECM extract composition. Surface characterization was performed using scanning electron microscopy and atomic force microscopy. We compared pECM with conventional coatings, including gelatin and non-coated controls, using morphological analysis, WST-1 assay, cell cycle analysis, and gene/protein expression of SSC markers. pECM promoted larger, well-defined pSSC colonies and enhanced stemness-related marker expression, including PGP9.5, Thy-1, PLZF, GFRA1, NANOG, and VASA. Additionally, pECM facilitated active pSSC proliferation while suppressing feeder overgrowth, contributing to a stable and functional co-culture environment. Conversely, gelatin supported early feeder proliferation but led to growth saturation, whereas N/C showed delayed attachment and reduced viability. These findings suggest that pECM mimics the native SSC niche and improves pSSC culture. The dual function of pECM in regulating feeder behavior and enhancing pSSC maintenance highlights its potential as a biomaterial for species lacking established feeder-free protocols.

## 1. Introduction

Spermatogonial stem cells (SSCs) are essential for studies on spermatogenesis and reproductive biology, the production of transgenic animals, and development of infertility treatments [1]. In addition to their biological role, SSCs hold considerable practical value. Their in vitro culture and manipulation enable applications such as fertility preservation, production of genetically modified animals, and accelerated breeding of livestock and other animals with special purposes. At present, the preservation and dissemination of elite genetic traits in livestock are largely achieved through DNA cloning, which is technically demanding and costly, or through the use of superior sires via natural mating and artificial insemination. However, the establishment of reliable SSC culture systems could provide a more cost-effective and scalable alternative, as SSCs can serve as renewable sources of germ cells. If functional spermatozoa can be consistently generated from SSCs, this approach would overcome the spatial and temporal constraints inherent to current breeding strategies, facilitating the preservation of valuable genetic resources and enabling more flexible and efficient animal breeding practices. Thus, establishing robust SSC culture platforms has important implications not only for basic reproductive biology but also for translational and industrial applications.

SSC function is significantly influenced by signals from various extracellular matrix (ECM) components, such as laminin, collagen IV, and nidogen (also known as entactin), which constitute the basement membrane of seminiferous tubules [2,3]. These signals are crucial for mimicking the in vivo environment in vitro and represent an important area of study. Current porcine SSC (pSSC) culture systems commonly employ co-culture with testicular somatic cells, mainly Sertoli cells, as feeder cells [4]. The ECM interactions provided to Sertoli cells strongly influence their characteristics and, in turn, affect the development of co-cultured SSCs [5]. Sertoli cells produce various ECM proteins that are essential for cellular functions [6]. Additionally, when each substance is applied individually, collagen IV promotes SSC differentiation, whereas laminin supports maintenance of the undifferentiated state [3].

Among the various ECM proteins used in in vitro culture, gelatin, derived from collagen degradation, is widely used as a coating material because it is cost-effective and highly biocompatible owing to its broad availability from diverse sources [7]. In particular, gelatin coating is a general condition in pSSC culture [8,9,10]. Although gelatin coatings offer practical advantages, their limited ability to replicate the native ECM underscores the need for improved biomimetic alternatives. In conventional gelatin-coated culture systems, the rapid proliferation of feeder cells necessitates frequent subculturing and a continuous supply of fresh feeder cells [4]. Moreover, during extended passages, SSC colonies fail to remain round and compact, eventually leading to their disappearance [4]. To effectively maintain and proliferate pSSCs, the coating material must facilitate the movement of feeder cells while simultaneously regulating their growth [8]. This highlights the need to develop new coating materials with specific properties.

Efforts to improve conventional coatings have involved the direct incorporation of the complex ECM composition of the natural environment into stem cell culture systems [11,12]. Porcine feet contain various ECM-rich tissues including skin, muscle, and cartilage making them a promising source of structural and functional ECM components [13,14]. Although porcine feet are occasionally used as food, processed into animal feed, or used as raw materials for gelatin production [15], a significant portion is still discarded in large quantities [16]. This underutilized resource has been identified as an ideal material for ECM extraction to enhance its value and promote sustainable reuse. We selected this tissue for hot-water extraction, which enables large-scale protein isolation while preserving key functional motifs [17,18,19], despite partial thermal denaturation. These features suggest the potential of porcine foot-derived ECM (pECM) as a supportive substrate for SSC culture.

This study aimed to evaluate the potential of pECM as an alternative ECM coating for pSSC culture. We hypothesized that pECM provides a more supportive microenvironment for pSSC and Sertoli cell proliferation and interaction. To test this, we compared pECM with gelatin and non-coated (N/C) controls in terms of colony formation, cell viability, and cell cycle progression under feeder co-culture conditions. In addition, to assess the preservation of SSC characteristics, we examined the expression of key undifferentiated and germline stem cell markers, including PGP9.5, Thy-1, PLZF, GFRA1, NANOG, VASA, and CD9, at both the mRNA and protein levels. These markers were selected to evaluate stemness maintenance, germline identity, and SSC–feeder interactions under different ECM conditions.

## 2. Results

### 2.1. Molecular Weight Distribution and Composition of pECM

**Figure 1:**
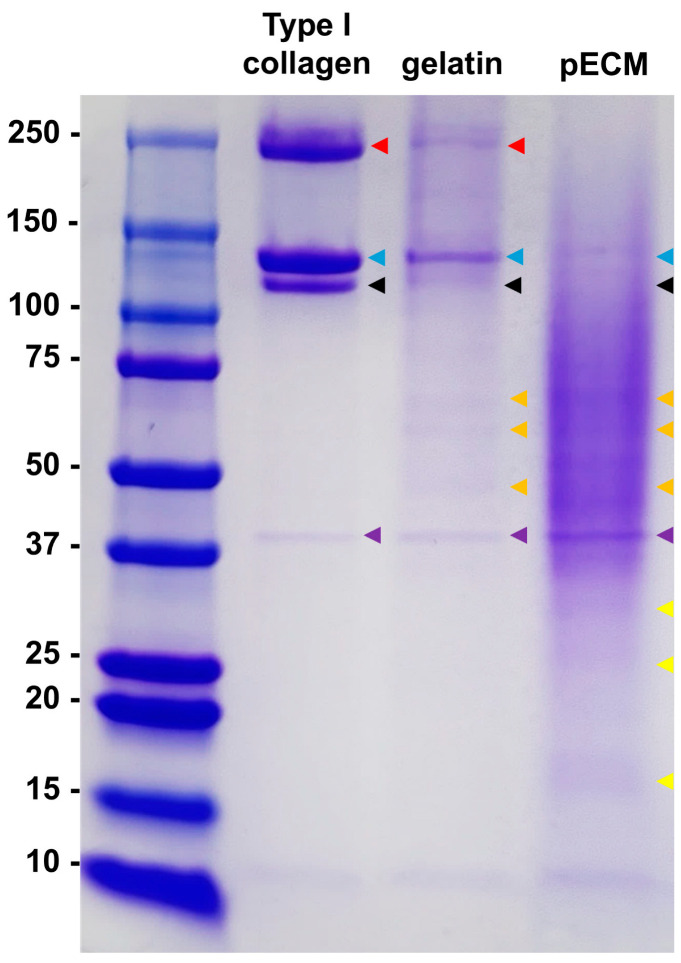
Sodium dodecyl sulfate-polyacrylamide gel electrophoresis analysis of Type I collagen, gelatin, and porcine extracellular matrix (pECM) (Coomassie blue staining). The arrows indicate major bands as follows: red, collagen β-chain; blue, α1-chain; black, α2-chain; purple, ~40 kDa band; orange, proteins in the 37–100 kDa range; and yellow, proteins < 37 kDa.

The relative composition of the pECM extract was quantitatively assessed (Supplementary Figure S1). The extract consisted predominantly of protein (~88%), with minor quantities of fat, carbohydrates, and ash. The molecular weight distribution of the extracted pECM was further analyzed using sodium dodecyl sulfate-polyacrylamide gel electrophoresis (Figure 1). Collagen β-chains (red arrows) were observed in Type I collagen and gelatin, whereas collagen α1-chains (blue arrows) and α2-chains (black arrows) were observed in all three samples (Type I collagen, gelatin, and pECM). In addition, a band at approximately 40 kDa (purple arrows), which was presumed to be a degraded form of collagen, was observed in all three samples. In gelatin and pECM, various bands were detected in the 37–100 kDa range (orange arrows), which were absent in the collagen sample. However, these bands were more prominent in pECM than in gelatin. Additionally, numerous proteins < 37 kDa (yellow arrows) were observed in the pECM but were absent in collagen or gelatin. In pECM, a diffuse staining pattern was prominent rather than distinct band formation.

### 2.2. Surface Roughness of Coating Materials

**Figure 2:**
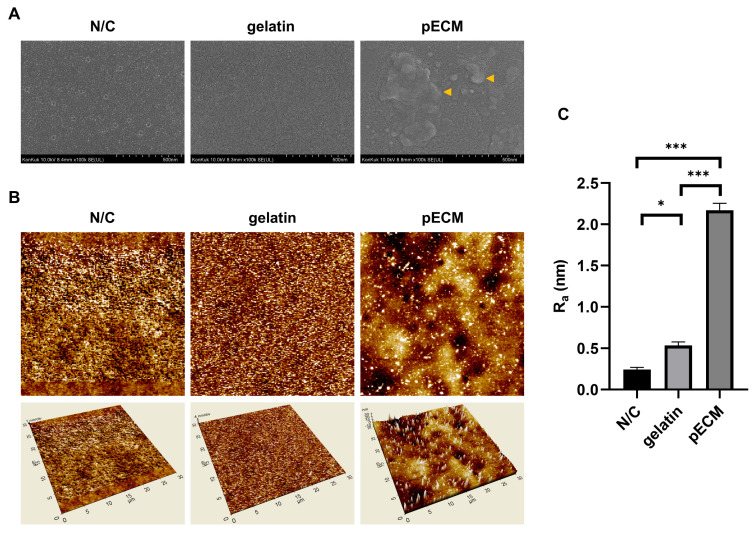
Surface morphological features and roughness analysis under different coating conditions (non-coated (N/C), gelatin, and pECM). (**A**) Scanning electron microscopy images of N/C, gelatin, and pECM. Yellow arrows indicate nanoscale protrusions on the pECM surface. (**B**) Representative atomic force microscopy 2D/3D images of each coating. (**C**) Quantitative surface roughness (R_a_). Data are shown as mean ± standard error of the mean (SEM) (*n* = 4); * *p* < 0.05, *** *p* < 0.001.

In the scanning electron microscopy images (Figure 2A), no notable surface structures were observed on the N/C plate, which remained relatively flat. In contrast, the gelatin-coated surface exhibited fine, groove-like microstructures. Although the pECM-coated plate was dried under similar conditions, these groove-like patterns were not observed. Instead, scattered surface protrusions were detected, which are presumed to be protein-derived features specific to pECM (yellow arrows).

To evaluate the nanoscale surface topography of each coating condition, atomic force microscopy (AFM) was performed on N/C, gelatin, and pECM-coated plates (Figure 2B). The uncoated N/C surface exhibited a relatively smooth morphology with minimal elevation, whereas the gelatin-coated surface exhibited slightly increased surface irregularities. In contrast, the pECM-coated surface displayed a heterogeneous and rugged topography with pronounced nanoscale protrusions.

Quantitative analysis of average surface roughness (R_a_) revealed that pECM-coated plates had significantly higher R_a_ values (2.169 ± 0.086 nm) than those of gelatin (0.534 ± 0.045 nm) and N/C (0.242 ± 0.027 nm) conditions (Figure 2C).

### 2.3. Comparison of Morphology, Proliferation, and Alkaline Phosphatase (AP) Activity of Cells Under Different Coating Conditions

**Figure 3:**
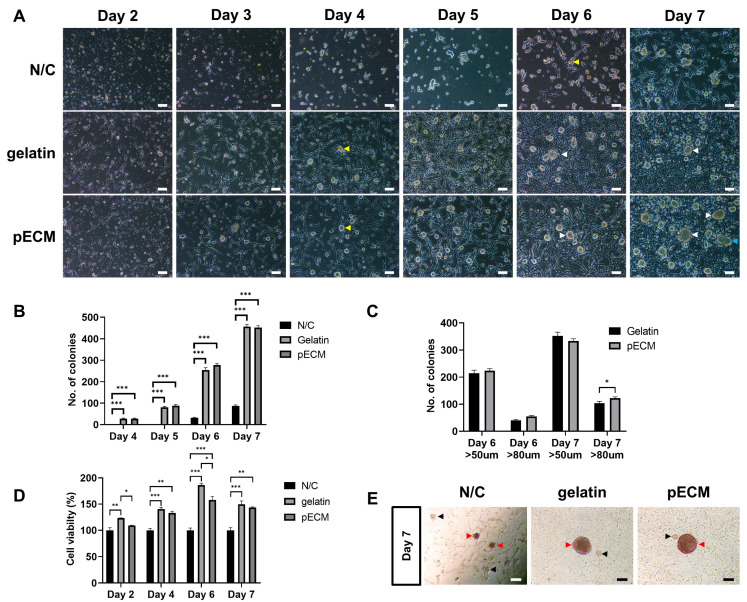
Characterization of porcine total testicular cells under different coating conditions. (**A**) Phase-contrast images from days 2–7. The arrows indicate the following: yellow, colonies > 50 µm; white, colonies > 80 µm; and blue, colony fusion. (**B**) Total number of colonies are represented as counts/100 mm^2^ on days 4–7 (*n* = 12). (**C**) Number of colonies classified by size (>50 µm and >80 µm) are represented as counts/100 mm^2^ on days 6–7 (*n* = 12). (**D**) Cell viability assessed using WST-1 assay (*n* = 3). (**E**) Alkaline phosphatase (AP) staining of colonies on day 7; red arrows indicate AP-positive colonies and black arrows indicate AP-negative clusters. * Scale bars = 100 μm. Data are presented as mean ± SEM. * *p* < 0.05, ** *p* < 0.01, *** *p* < 0.001.

The morphology and development of porcine total testicular cells (pTTCs) were compared under three different coating conditions (N/C, gelatin, and pECM) from days 2 to 7 (Figure 3). In all groups, SSCs were co-cultured with feeder cells, and only the surface coating differed among the conditions: the N/C condition served as the control, gelatin was used as a commonly applied coating, and pECM was introduced as the experimental coating. Throughout the culture period, the N/C condition exhibited poor adherent testicular somatic cell (pFeeder) attachment and inadequate cell spreading (Figure 3A). In contrast, under both gelatin and pECM conditions, most cells were attached by day 2, and the pFeeders were well spread out. During the culture period, noticeable differences in pFeeder growth were observed, particularly on days 4, 5, and 6. On day 4, colonies with a diameter > 50 µm (yellow arrows) were observed for the first time under both gelatin and pECM conditions, indicating the initial formation of colonies in these environments. Conversely, the N/C did not exhibit colonies attached to pFeeders. By day 6, colonies with diameters > 80 µm (white arrows) were observed under both gelatin and pECM conditions. In contrast, in N/C conditions, colonies with diameters > 50 µm, which showed a clear boundary with pFeeders, were observed for the first time (yellow arrows). On day 7, the colonies cultured on pECM tended to have larger diameters than those cultured under the other conditions. Additionally, in the pECM, colony fusion was more frequently observed on day 7 (blue arrows, Figure 3A).

Colony counting results showed a steady increase in the number of colonies from days 4 to 7 under the three different conditions (Figure 3B). Although gelatin exhibited a slightly higher total number of colonies on day 7, pECM supported a greater proportion of larger colonies (>80 µm) (Figure 3C). Across all time points, colony numbers were consistently lowest under the N/C condition. Cell viability also showed a consistent pattern across the culture period: gelatin showed the highest viability, followed by pECM, with N/C displaying the lowest values at all time points (Figure 3D).

AP staining revealed the presence of AP-positive colonies under all three conditions, although the relative colony sizes varied. When comparing colonies of similar sizes, those cultured on pECM exhibited relatively stronger AP staining intensity than those cultured on gelatin (Figure 3E). AP-positive colonies (red arrows) and AP-negative colony-like clusters (black arrows) are observed in all groups. The AP staining intensity is stronger in the pECM. Together, colony formation and AP staining served as functional assays to confirm SSC stemness, reflecting their capacity for self-renewal and maintenance of an undifferentiated state.

### 2.4. Cell Cycle Analysis of pSSC Colonies and pFeeders Under Different Coating Conditions

**Figure 4:**
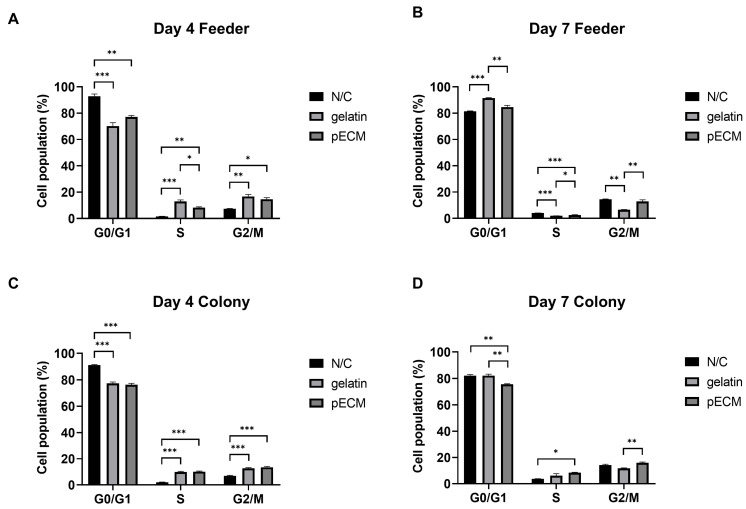
Cell cycle analysis of adherent testicular somatic cells (pFeeders) and porcine spermatogonial stem cell (pSSC) colonies under different coating conditions on days 4 and 7. (**A**) Cell cycle distribution of pFeeders on day 4. (**B**) Cell cycle distribution of pFeeders on day 7. (**C**) Cell cycle distribution of pSSC colonies on day 4. (**D**) Cell cycle distribution of pSSC colonies on day 7. Data are presented as mean ± SEM (*n* = 3). * *p* < 0.05, ** *p* < 0.01, *** *p* < 0.001.

The cell cycle distributions of both pFeeders and pSSC colonies were analyzed on days 4 and 7 under the three different coating conditions (Figure 4), and the representative histograms used for the estimation of phase proportions are provided in Supplementary Figure S2.

On day 4, pFeeders under N/C conditions showed a significantly higher proportion of G0/G1 phase than those in the other groups, whereas both gelatin and pECM coatings increased the proportion of S and G2/M phases (Figure 4A). Notably, the S phase was significantly higher under pECM than under gelatin conditions. On day 7, pFeeders under gelatin conditions showed the highest G0/G1 phase, while the S phase was most elevated in the N/C group and significantly higher in pECM than in gelatin. In the G2/M phase, both the N/C and pECM groups showed higher proportions than the gelatin group (Figure 4B).

On day 4, pSSC colonies under N/C conditions showed the highest G0/G1 phase, whereas both gelatin and pECM significantly increased the proportions of S and G2/M phases compared to N/C (Figure 4C). On day 7, the G0/G1 phase remained significantly higher in the N/C and gelatin groups, whereas the S phase was significantly elevated in the pECM group compared to that in the N/C group, with gelatin showing an intermediate level. In the G2/M phase, pECM exhibited the highest proportion among the three conditions (Figure 4D).

### 2.5. Gene and Protein Expression Analysis of Day 7 Colonies

**Figure 5:**
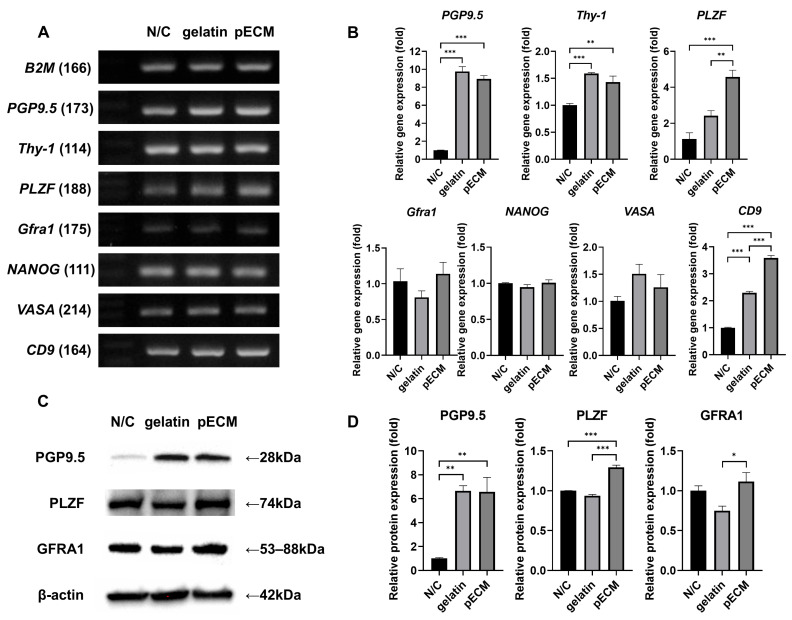
Gene and protein expression analysis of pSSC colonies on day 7 under the three coating conditions. (**A**) Reverse transcription-polymerase chain reaction (RT-PCR) analysis of SSC-related markers. Undifferentiated spermatogonia markers: *PGP9.5*, *Thy-1*, and *PLZF*. SSC stemness marker: *GFRA1*. Pluripotent marker: *NANOG*. Germline marker: *VASA*. SSC marker: *CD9*. Housekeeping control: *B2M*. (**B**) Quantitative PCR (qPCR) analysis of the same markers. (**C**) Western blot analysis of PGP9.5, PLZF, and GFRA1 protein expression, with β-actin used as the loading control. (**D**) Quantification of relative protein expression normalized to β-actin. Data are presented as mean ± SEM (*n* = 4). * *p* < 0.05, ** *p* < 0.01, *** *p* < 0.001.

To evaluate the expression of SSC-related genes and proteins in the early stages of culture, colonies cultured under all three conditions were harvested on day 7 for gene and protein expression analyses (Figure 5). Visual inspection was used to confirm the separation of pSSC colonies prior to RNA and protein sampling (Supplementary Figure S3A). WT1, a Sertoli cell-specific marker, was used to assess feeder contamination, and its markedly lower expression in colony samples confirmed sufficient separation from feeder cells for gene expression analysis (Supplementary Figure S3B).

In the day 7 colonies, reverse transcription-polymerase chain reaction (RT-PCR) analysis detected bands corresponding to *B2M*, *PGP9.5*, *Thy-1*, *PLZF*, *Gfra1*, *NANOG*, *VASA*, and *CD9* (Figure 5A). These markers included undifferentiated spermatogonia markers (*PGP9.5*, *Thy-1*, and *PLZF*), an SSC stemness marker (*Gfra1*), a pluripotent marker (*NANOG*), a germline marker (*VASA*), and an additional SSC marker (*CD9*). For *PGP9.5*, the band intensity was stronger in gelatin and pECM than in N/C. Both *PLZF* and *CD9* showed a gradual increase in band intensity from N/C to gelatin to pECM, with the most prominent bands detected in pECM.

Quantitative PCR (qPCR) analysis confirmed that *PGP9.5* and *Thy-1* were significantly upregulated in gelatin and pECM compared with N/C, whereas *PLZF* expression was highest in pECM (Figure 5B). *Gfra1*, *NANOG*, and *VASA* showed no significant differences among the groups. *CD9* expression was highest in pECM, intermediate in gelatin, and lowest in N/C.

Western blot analysis confirmed the presence of PGP9.5, PLZF, and GFRA1 in all groups (Figure 5C). When normalized to β-actin, PGP9.5 and PLZF showed expression patterns consistent with the qPCR results, with significantly higher levels in gelatin and pECM than in N/C, and the highest expression was detected in pECM (Figure 5D). GFRA1 exhibited a similar pattern, with significantly higher expression in pECM than in gelatin. To further confirm that the observed protein expression originated from the colonies, immunocytochemistry was performed (Supplementary Figure S4), and all three markers (PGP9.5, PLZF, and GFRA1) were detected within the colonies.

### 2.6. Gene and Protein Expression Analysis of Day 13 Colonies

**Figure 6:**
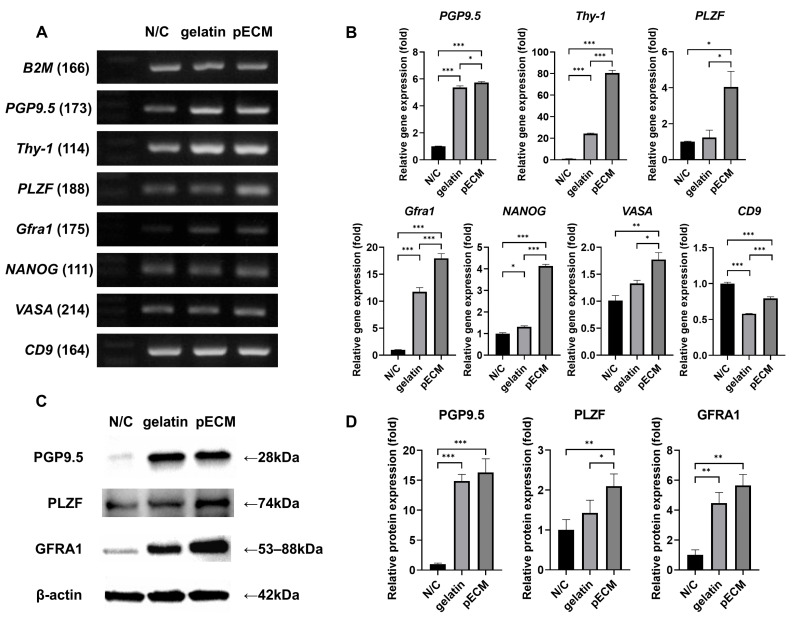
Gene and protein expression analysis of pSSC colonies on day 13 under the three coating conditions. (**A**) RT-PCR analysis of SSC-related markers (same markers as in Figure 5). (**B**) qPCR analysis of the same markers. (**C**) Western blot analysis of PGP9.5, PLZF, and GFRA1 protein expression, with β-actin used as the loading control. (**D**) Quantification of relative protein expression normalized to β-actin. Data are presented as mean ± SEM (*n* = 4). * *p* < 0.05, ** *p* < 0.01, *** *p* < 0.001.

As for day 7, gene and protein expression analyses were conducted on the colonies cultured until day 13 to assess changes after subculture (Figure 6). RT-PCR analysis confirmed that all markers were expressed in day 13 colonies under all three conditions (Figure 6A). Undifferentiated spermatogonia markers (*PGP9.5*, *Thy-1*, and *PLZF*) and the SSC stemness marker (*Gfra1*) showed noticeably weaker bands in N/C compared to gelatin and pECM, whereas *NANOG*, *VASA*, and *CD9* exhibited comparable expression across the groups.

Quantitative differences were further assessed using qPCR (Figure 6B). qPCR analysis revealed that *PGP9.5*, *Thy-1*, *Gfra1*, and *NANOG* exhibited a clear pattern of upregulation (N/C < gelatin < pECM), with the highest expression under pECM conditions. In contrast, *PLZF* and *VASA* showed no significant difference between N/C and gelatin, but were most strongly expressed in pECM. *CD9* displayed the opposite trend, with the highest expression in N/C, intermediate expression in pECM, and the lowest expression in gelatin.

Western blotting was performed to evaluate the protein expression of SSC markers on day 13 (Figure 6C). PGP9.5 expression was lowest in N/C and significantly higher in both gelatin and pECM, consistent with the day 7 results (Figure 6D). PLZF also showed the highest expression in pECM, with significantly greater levels than in N/C and gelatin. GFRA1 expression followed a similar pattern, being significantly higher in gelatin and pECM than in N/C.

## 3. Discussion

### 3.1. Implications of pECM for SSC Culture Systems

The ECM is composed of various proteins that provide essential biochemical signals to cells and play crucial roles in regulating key cellular behaviors, including adhesion, migration, proliferation, and differentiation. ECM-based materials have been widely applied in tissue engineering and regenerative medicine, not only as scaffolds but also in the form of hydrogels that can be combined with bioinks for tissue regeneration and transplantation [20].

In the context of SSC culture, feeder-free approaches have been explored using various matrix substitutes. For example, Matrigel supports SSC expansion in rodents [21], and the long-term culture of human SSEA-4–positive SSCs [22]. In addition, single ECM proteins such as laminin and collagen have been applied individually in human SSC cultures, where they either promote the maintenance of the undifferentiated state or induce differentiation [23]. More physiologically relevant outcomes have been reported with homologous decellularized testis-derived ECM. In rodents, such scaffolds support not only SSC proliferation but also differentiation into spermatocytes and spermatids, demonstrating their potential as in vitro platforms for spermatogenesis [24]. Additionally, a recent study reported that a testis-derived ECM hydrogel, when combined with Matrigel, provided an efficient feeder-free 3D culture platform that promoted both the proliferation and differentiation of mouse SSCs [25]. In humans, testis-derived ECM enhances SSC survival and maintenance in vitro under feeder-free conditions [26]. More recently, when combined with testicular cells and platelet-rich plasma, decellularized human testicular scaffolds promoted SSC differentiation rather than maintenance [27]. Collectively, these findings indicate that homologous-derived ECM provides superior outcomes compared to Matrigel or single-protein coatings, emphasizing its importance in developing physiologically relevant culture systems.

In contrast to rodents and humans, studies on pigs remain scarce. Previous studies have shown that pSSC colonies arise primarily through interactions with Sertoli cells, highlighting the technical difficulty of establishing feeder-free conditions in this species [9]. Based on these limitations, we applied pECM coatings within feeder co-culture systems to evaluate whether a tissue-derived ECM could improve the balance between feeder cell regulation and SSC maintenance. This approach offers a novel and less explored perspective that extends the existing knowledge from rodent and human models to the porcine system.

Despite recent advances in synthetic scaffolds and 3D culture systems, these approaches have largely focused on promoting differentiation or functional maturation rather than sustaining an undifferentiated state [27]. Such systems may offer valuable platforms for downstream application; however, the reliable procurement of undifferentiated SSCs remains a prerequisite. In this context, 2D co-culture systems continue to represent the most efficient strategy for expanding undifferentiated SSC populations. Our findings highlight that pECM, by modulating feeder cell behavior and supporting SSC marker expression, provides a supportive niche within this framework. Thus, the use of porcine-derived ECM contributes to the existing knowledge by bridging the gap between conventional feeder-dependent 2D cultures and advanced scaffold-based approaches, ensuring a stable source of undifferentiated SSCs for subsequent translational applications.

### 3.2. Microenvironment Patterns for pSSC Culture

In this study, we prepared pECM derived from the complex composition of natural ECM and investigated the effect of pECM coating on the in vitro culture of pSSCs. Porcine feet are composed of skin, muscle, and cartilage, which are rich in basement membrane components such as collagen, laminin, and nidogen, as well as proteoglycans and glycosaminoglycans (GAGs) [14,28]. The extraction of these proteins was supported by the presence of various protein bands, likely reflecting differences in their molecular weights [29]. Given this composition, the hydrolyzed ECM extracted from porcine feet may exert beneficial effects in stem cell culture. For example, hydrolyzed porcine skin collagen produced by hydrothermal processing exhibits strong antioxidant activity in human fibroblasts [17]. Moreover, hydrolyzed collagen increases the expression of pro-collagen-1α in both human fibroblasts and keratinocytes [30]. Covalently bound RGD (Arg–Gly–Asp, originally identified in fibronectin and also found in several other ECM proteins) and YIGSR (Tyr–Ile–Gly–Ser–Arg, derived from the laminin β1 chain) peptides alone are sufficient to facilitate cellular attachment to an extent similar to that of their respective native ECM proteins [31]. Accordingly, these motifs are likely to remain functional in pECM despite heat denaturation during the extraction process, and if preserved, they may exert similar bioactivities to support cell adhesion and behavior. Taken together, these findings indicate that pECM derived from porcine feet contains a richer and more diverse set of ECM proteins than gelatin, including both structural proteins and functional motifs. This complex composition may provide a more physiologically relevant microenvironment that better mimics native ECM characteristics, offering potential advantages for supporting SSC cultures.

Scanning electron microscopy revealed distinct surface structures between the gelatin- and pECM-coated plates. Groove-like surface features were observed on the gelatin-coated plates, which were presumed to be the result of the drying and shrinkage of the gelatin film. In contrast, such patterns were absent on pECM-coated plates, which instead exhibited scattered nanoscale protrusions, likely arising from the complex composition of ECM proteins. These morphological differences may stem from the distinct physical properties of the coating materials. The pECM layer contains a diverse mixture of proteins with relatively low average molecular weights, which tend to adsorb onto the surface without forming film-like layers [32]. In contrast, gelatin predominantly consists of large proteins that spread more continuously across the surface and are prone to shrinkage upon drying, leading to the formation of groove-like surface patterns.

Surface roughness is an important factor that influences cell adhesion and behavior. Previous studies have explored how surface roughness at the nano- to microscale level can modulate cell behavior in various cell types. For instance, MG-63 osteoblast-like cells exhibit reduced proliferation but enhanced motility on rough surfaces compared to smooth surfaces [33,34]. Similarly, studies using chick embryo vascular and corneal explants have reported that increased surface roughness enhances cell migration, while having little effect on proliferation or cell viability [35]. Furthermore, human mesenchymal stem cells (hMSCs) exhibit altered adhesion, proliferation, and differentiation depending on nanoscale roughness, as demonstrated in studies on thermoplastics [36]. In this context, the significantly higher surface roughness of pECM compared to that of gelatin and N/C may have contributed to the enhanced colony formation and moderated feeder cell growth observed under pECM conditions. Thus, while the unique protein composition of pECM is likely the dominant driver, topographical cues such as surface roughness cannot be entirely excluded as contributing factors to the distinct cellular behaviors observed.

### 3.3. Regulation of Cellular Dynamics in pSSCs and Feeder Cells by pECM

Among the three coating conditions, pECM showed relatively stronger AP staining than gelatin, and exhibited the highest expression of multiple markers associated with stemness or the undifferentiated state, including PGP9.5, PLZF, GFRA1, NANOG, and VASA. These results indicate that pECM provides a more favorable microenvironment for maintaining the undifferentiated state of pSSCs during in vitro culture. In support of this, a previous study demonstrated that the use of a homologous composite ECM provides the most favorable environment for the maintenance and expansion of human SSCs under feeder-free conditions [26]. In addition, in the co-culture of human SSCs with Sertoli cells, the collagen-coated environment resulted in a greater number and larger diameter of colonies compared to the N/Cs condition [37].

Furthermore, the morphological characteristics and cell cycle progression also demonstrated clear differences across the coating conditions, further reinforcing the effect of the ECM environment on pSSC behavior. In particular, colony counting and cell cycle analysis showed that pECM supported a higher proportion of large colonies and enhanced pSSC proliferation during in vitro culture. These findings are consistent with previous results showing that larger stem cell colonies exhibit higher local cell densities, which in turn lead to increased levels of bone morphogenetic protein antagonists and enhanced self-renewal capacity [38]. These results indicate that pECM not only supports colony formation but also promotes pSSC proliferation during in vitro culture. 

Feeder cells are generally required to maintain the stemness of SSCs, and Sertoli cells are mainly used as feeders in pSSC cultures [4,39]. Sertoli cells influence the self-renewal and differentiation of pSSCs during co-culture, primarily through exosomes and various cell-to-cell interaction mechanisms [40]. Based on the morphological observations of feeder cells, it can be inferred that pECM enhances feeder cell migration. Throughout the culture period from day 2 to day 7, the N/C condition not only exhibited slower overall attachment, but also less prominent extended filopodia. In contrast, under both pECM and gelatin conditions, uniform attachment of pFeeders was observed by day 2, and filopodia were well extended. Sertoli cells act as carriers for pSSCs, facilitating their migration to colony-forming regions while remaining attached to pSSCs [8]. The observed colony formation outcomes suggest that pECM may provide pFeeders with greater mobility than gelatin or N/C. This interpretation is further supported by the observation that extended filopodia, which are typically seen in motile cells, were less prominent under N/C conditions but were well-developed under pECM conditions [41]. A previous study showed that if cell–substrate adhesion is either too strong or too weak, cell movement is inhibited, indicating that an optimal level of adhesion is required for efficient migration. This supports the interpretation that pECM offers an appropriate level of adhesion to facilitate Sertoli cell migration [42].

Furthermore, pECM appeared to suppress excessive proliferation of pFeeders. While the WST-1 assay provided useful information on overall cell viability, we acknowledge that this method has inherent limitations in 3D colony settings [43], as it mainly reflects the activity of surface-exposed cells and is strongly influenced by feeder proliferation rather than pSSC growth. Therefore, a combined analysis with cell cycle data was essential to distinguish between feeder expansion and SSC contribution more accurately. Based on this combined analysis, although gelatin showed the highest overall viability, this was likely attributable to feeder cell overgrowth rather than pSSC expansion. In contrast, pECM supported more balanced growth, with greater pSSC contribution to total cell viability. The N/C condition exhibited limited initial cell attachment and viability, likely due to the absence of ECM cues, and delayed feeder cell growth was observed over time. This interpretation is supported by previous findings showing that inadequate ECM attachment reduces cyclin D/Cdk4–6 activity, and that high cell density induces Hippo pathway activation, which suppresses cyclin D transcription, ultimately leading to G1 arrest [44]. Taken together, these findings suggest that, in contrast to gelatin, pECM regulates pFeeder proliferation more effectively while preventing excessive overgrowth. More broadly, the ability of pECM to simultaneously promote feeder cell migration and modulate proliferation offers a functional advantage in feeder-dependent culture systems, particularly for maintaining pSSCs that require periodic subculturing once feeders reach confluence.

### 3.4. Modulation of SSC-Associated Marker Expression by pECM

*PGP9.5* is a strong marker of undifferentiated germ cells in several species. In addition, *PLZF* is a key regulator of spermatogonial self-renewal in mice and plays a crucial role in SSC maintenance [45,46]. *GFRA1* is a marker of SSCs in mice and functions as a receptor for glial cell line-derived neurotrophic factor (GDNF), which is essential for maintaining SSC stemness [47]. *NANOG* and *Thy-1* are widely recognized pluripotency markers [48,49]. Their high expression in pECM samples suggests that pSSCs retain their stemness during the culture process. The germline cell markers *VASA* and *CD9* have also been used to obtain information on a broad range of germline stem cells [50,51]. In agreement with these previous studies, *PGP9.5*, *Thy-1*, and *GFRA1* showed clear expression differences between the N/C and coated conditions, with pECM and gelatin showing higher expression levels in the present study. Notably, *PGP9.5* exhibited the largest fold change among all markers on day 7, suggesting its potential as a sensitive early indicator of the effectiveness of the extracellular environment in supporting SSC identity. By day 13, all analyzed markers (*PGP9.5*, *Thy-1*, *PLZF*, *GFRA1*, *NANOG*, and *VASA*) were highly expressed in the pECM condition, indicating that pECM better supports the long-term maintenance of stemness-related and germline-associated genes in pSSCs. The time-dependent divergence in the expression of SSC markers suggests that pECM more closely mimics the biochemical properties of the native SSC niche. This pECM not only supported the early expression of SSC markers but also maintained and enhanced their expression during prolonged culture. The fact that *PGP9.5* and *PLZF* are selectively and strongly expressed in SSCs located near the basement membrane further supports this conclusion [52]. Additionally, the combined analysis of colony morphology and marker expression aligned with previous findings, showing that larger, well-defined colonies are indicative of an undifferentiated state [53].

However, *CD9* exhibited a distinct expression pattern. Rather than following the general trend of progressive upregulation in pECM, its expression appeared to vary depending on the type of cell–cell interaction present. As a molecule involved in cell–cell adhesion, CD9 modulates the interactions between integrin α5β1 and ADAM17, thereby influencing ECM binding and the retention of adhesion molecules on the cell surface [54]. These adhesion-related functions suggest that *CD9* expression may be adaptively regulated in response to different pSSC–pFeeder and pSSC–pSSC interactions, depending on the coating environment. This functional specificity may explain its deviation from the expression profiles of other SSC-associated genes. Further studies are needed to investigate these factors and elucidate their regulatory mechanisms at the protein level.

### 3.5. Further Applications of pECM

Selecting a suitable ECM for cell culture is a fundamental requirement in 3D culture and tissue engineering, which has led to the development and evaluation of various ECM candidates [55]. Efforts to identify suitable ECMs have led to various discoveries, including the use of decellularized tissues and Matrigel [12,56]. Compared to these ECMs, pECM offers advantages in terms of cost-effectiveness and ease of use. In the current approach of mimicking the biological environment through a combination of various materials [57], pECM can serve as a provider of diverse motifs. The effects of pECM observed in 2D cultures, including the promotion of pSSC proliferation and regulation of feeder cell growth, may also be applicable to tissue regeneration and organoid research through similar mechanisms. This concept is supported by a recent study demonstrating the generation of organized porcine testicular organoids in solubilized hydrogels derived from decellularized ECM [58], highlighting the potential of ECM-based systems to recreate complex testicular microenvironments.

In addition, feeder-free culture systems using Matrigel have been established in mice [21], and feeder-free conditions are actively being explored in various stem cell studies to improve standardization and reproducibility [26]. Previous studies have demonstrated that conjugating short laminin-derived peptides with hydrogels improves neural stem cell growth [59]. Similarly, the combinatory use of pECM with other ECM components, which contain relatively short peptide motifs, may facilitate the development of a feeder-free culture system for pSSCs, which has yet to be established. Furthermore, given that pECM contains diverse ECM motifs, it also holds potential for application in the 3D culture of pSSCs with scaffolds.

In pig production, only a small proportion of males are retained as breeding boars, whereas most are castrated early, making the maintenance and selection of elite sires costly. Stable SSC culture and transplantation techniques could accelerate genetic improvement and reduce costs by enabling faster breeding cycles. Moreover, the ultimate application of SSC culture lies in in vitro spermatogenesis or germ cell production through transplantation, as demonstrated in DAZL knockout pigs that generated functional germ cells after receiving donor SSCs [60]. Such approaches could also allow non-breeding males to be converted into functional germline donors, thereby expanding genetic diversity and preserving valuable resources. The broader application of SSC technologies will inevitably require culture environments that are both productive and cost-effective, and the pECM investigated in this study has the potential to contribute to meeting these requirements.

### 3.6. Limitations

While this study demonstrated that pECM supports the maintenance of pSSCs, some limitations must be acknowledged. First, a detailed characterization of the polypeptide components of pECM is required to better understand its functional properties and facilitate its large-scale production and broader application. Second, the relatively small sample size may limit the generalizability of our findings. Third, the underlying molecular mechanisms remain unclear, and future studies should employ transcriptomic approaches such as RNA sequencing to identify key signaling pathways. Fourth, investigating gene expression changes in pFeeders cultured on pECM would help clarify its role in regulating the stem cell niche. Finally, in vivo transplantation experiments are essential to validate whether pSSCs maintained under these conditions can contribute to spermatogenesis. Although such experiments were not conducted in this study, previous studies using the same platform have demonstrated that cells characterized by SSC markers, AP activity, and colony-forming ability could successfully engraft after transplantation into mouse testes, providing indirect support for the functional relevance of these characteristics [4,61].

## 4. Materials and Methods

### 4.1. Extraction of pECM from Porcine Foot Tissue

Porcine foot tissue, pre-trimmed to remove hooves and sectioned into suitable sizes for handling, was sourced from a local slaughterhouse from YLD (Yorkshire × Landrace × Duroc) crossbred pigs. For ECM preparation, both forefoot and hindfoot tissues from the same animals were used in combination to reduce variability across different anatomical sites. All tissues were stored at −70 °C until further processing. First, 600 g of tissue was immersed in a sufficient volume of distilled water for 4 h to remove blood and other impurities. The tissue was then finely ground and washed in 3 L distilled water for 1 h with agitation, and this step was repeated twice to remove further impurities [62,63]. Following the complete removal of residual moisture, 3 L of distilled water at 100 °C was added to the tissue and maintained at this temperature for 10 min to further eliminate blood and impurities, including cellular components. After removing the water containing residue and performing additional washing, another 3 L of distilled water was added, and the tissue was subjected to prolonged heating at 100 °C for 16 h under atmospheric pressure, with the water replenished as needed throughout the process. The resulting solution was sequentially filtered through 100-mesh and 300-mesh filters to enhance purity. After filtration, the extract was centrifuged at 10,000× *g* for 1 h at room temperature (RT), and the pellet and upper oil layers were removed. The centrifugation step was repeated twice to obtain a refined solution. The final solution was dried at 60 °C, powdered, and stored at −20 °C until further use. This extraction process was independently repeated three times using tissues from different porcine individuals, and consistent yields were obtained.

### 4.2. Proximate Composition and Protein Analysis of pECM

Compositional analysis of the pECM extract (protein, fat, carbohydrate, and ash) was performed externally at the Korea Food Research Institute (Wanju, Republic of Korea), and the results are shown in Supplementary Figure S1.

The protein content of the pECM working solutions was measured using a Pierce BCA Protein Assay Kit (VK312556, Thermo Fisher Scientific, Waltham, MA, USA), with bovine serum albumin (BSA) as the standard. The protein concentrations of the cell lysates prepared for Western blotting were also determined using the same kit. Thirty micrograms each of Type I collagen standard, gelatin, and pECM were separated using polyacrylamide gel electrophoresis using 4–20% Mini-PROTEAN^®^ TGX™ Precast Protein Gels (456-1095; Bio-Rad, Hercules, CA, USA), followed by staining with Coomassie Blue.

### 4.3. Isolation of pTTCs from Porcine Testes

pSSCs were derived as previously described, with some modifications [4]. Porcine testes were obtained from 5-day-old crossbred piglets from a local farm (Sam-Woo Breeding Farm, Yang Pyung, Republic of Korea). Decapsulated testes, seminiferous tubules, and interstitial tissues were dissected from the connective tissues. The mechanically dissected testes were digested with an equivalent volume (*v*/*v*) of enzyme A [0.5 mg/mL collagenase type IV (17104-019; Gibco, Carlsbad, CA, USA), 0.01 mg/mL DNase I (D5025; Sigma-Aldrich, St. Louis, MO, USA), 0.1 mg/mL soybean trypsin inhibitor (17075-029; Gibco), and 0.1 mg/mL hyaluronidase (H3506; Sigma-Aldrich)] at 37 °C for 15 min. The testes were washed with Dulbecco’s phosphate-buffered saline (DPBS; LB 001-02; Solbio, Suwon, Republic of Korea) by centrifugation at 650× *g* at RT. The dissected seminiferous tubules and interstitial tissue were treated with an equivalent volume (*v*/*v*) of enzyme B [5 mg/mL collagenase type IV, 0.01 mg/mL DNase I, and 0.1 mg/mL soybean trypsin inhibitor] at 37 °C for 15 min, followed by washing with DPBS via centrifugation at 650× *g* at RT. The testes cells were filtered using a 40 μm nylon mesh, and the red blood cells were removed using RBC lysis buffer (R7757; Sigma-Aldrich).

### 4.4. pSSC Culture on Different Coating Environments

To prepare a 1% (*w*/*v*) pECM solution, lyophilized pECM powder was dissolved in distilled water to a final concentration of 1% (*w*/*v*) and filtered using a 0.2 μm Minisart NML syringe filter (16534; Sartorius, Göttingen, Germany) to remove particulates and ensure sterility. The filtered solution was stored at 4 °C and used for plate coating within two weeks of preparation.

To prepare 0.1% (*w*/*v*) gelatin and 1% (*w*/*v*) pECM-coated plates, 400 µL of 0.1% gelatin (LS023-01) or 1% pECM solution was added to each well of 12-well plates, and 4 mL was added to 150 mm dishes and incubated at RT for 30 min. After incubation, the solutions were removed by suction, and the plates were air-dried under UV light for 30 min. Each plate was coated using the same procedure but with different coating solutions.

The in vitro pSSC culture method has been described previously [4]. Isolated pTTCs were seeded at a density of 2 × 10^5^ cells/well onto N/C, gelatin-coated, and pECM-coated wells of 12-well plates and incubated at 34 °C in 5% CO_2_ with Stempro-34 medium (10640-019; Gibco) supplemented with insulin-transferrin-selenium (ITS; 25 μg/mL, 100 μg/mL, and 30 nM; 41400-045; Gibco), 6 mg/mL D+ glucose (G6152; Sigma-Aldrich), 2 mM l-glutamine (25030; Gibco), 1 × non-essential amino acid solution (11140; Gibco), 1 × vitamin solution (11120; Gibco), 100 units/mL streptomycin-penicillin (15140; Gibco), 1 mM sodium pyruvate (11360; Gibco), 0.1 mM vitamin C (A4403; Sigma-Aldrich), 1 μg/mL lactic acid (L1750; Sigma-Aldrich), 30 ng/mL estradiol (E2758; Sigma-Aldrich), 60 ng/mL progesterone (P7756; Sigma-Aldrich), 0.2% BSA (BSA-100; Bovogen, Keilor East, VIC, Australia), knockout serum replacement (10828; Gibco), 10 ng/mL epidermal growth factor (Millipore, Billerica, MA, USA), 10 ng/mL basic fibroblast growth factor (AF-100-18B; Peprotech, Rocky Hill, NJ, USA), 10 ng/mL GDNF (512-GF-010; R&D Systems, Minneapolis, MN, USA), and 10^3^ U/mL leukemia inhibitory factor (ESG1170; Millipore) for the culture of pSSC. All experimental groups were maintained under feeder-dependent conditions in which SSCs were co-cultured with feeder cells. The only difference among the groups was the surface coating applied to the culture plates: N/C as the control, gelatin-coated, and pECM-coated.

During subculture, when pFeeders became confluent, the total cells containing pSSC colonies and feeder cells were collected using 0.25% trypsin-EDTA (25300-054; Gibco). The cells were suspended as single cells by treatment with 0.05% trypsin-EDTA, which was prepared by diluting 0.25% trypsin-EDTA (Gibco) with DPBS (Solbio) at a 1:5 ratio, at 37 °C for 5 min. Next, 2 × 10^7^ cells were seeded onto 0.1% (*w*/*v*) gelatin-coated 150 mm plates and incubated at 34 °C in 5% CO_2_ for 2 h. Subsequently, 1.5 × 10^5^ suspended cells and 0.5 × 10^5^ pFeeders were seeded onto either gelatin- or pECM-coated wells of new 12-well plates. The cells were sub-cultured in new plates with the same coating (pECM or gelatin) to maintain consistent culture conditions.

### 4.5. Surface Morphology of Coating Conditions Analyzed by Scanning Electron Microscopy

The surface morphologies of the N/C, gelatin, and pECM coatings were evaluated using field emission scanning electron microscopy (Hitachi SU8010, Hitachi High-Technologies, Tokyo, Japan) at an accelerating voltage of 10 kV and a magnification of ×100,000. To ensure a flat and uniform substrate for imaging, each coating solution, including the previously prepared 1% (*w*/*v*) pECM solution, was applied to sterile cover glasses using the same coating procedure as for the cell culture plates. Analyses were performed in quadruplicate (*n* = 4), and representative images are presented.

### 4.6. Surface Topography Analyzed by AFM

Surface roughness analysis was performed using AFM to evaluate the N/C, gelatin, and pECM coatings [64,65]. The measurements were conducted using an XE-100 AFM (Park Systems, Suwon, Republic of Korea) under ambient conditions (22 ± 2 °C, 25 ± 4% relative humidity). To ensure a flat imaging substrate, each coating solution was applied to sterile cover glasses using the same procedure as for the cell culture plates. AFM imaging was conducted in tapping mode with a scan area of 30 × 30 μm. Topographic images were acquired using optimized scanning parameters that were appropriate for each sample. All AFM measurements were conducted in quadruplicate (*n* = 4), and representative 2D and 3D images are shown.

### 4.7. WST-1 Cell Viability Assay

Cell proliferation assays were performed using an EZ-Cytox Viability Assay Kit (EZ1000; Dae-il Lab Services Co., Seoul, Republic of Korea). The pTTCs were seeded at a density of 2 × 10^5^ cells/well in N/C-, gelatin-, and pECM-coated 12-well culture plates. The assay reagent was prepared by dilution to a final concentration of 10% (*v*/*v*) in the culture medium. The existing medium was gently removed by suction, and the prepared reagent was added to the wells. The plates were incubated for 90 min on day 2, 60 min on day 4, 60 min on day 6, and 30 min on day 8. The supernatant from each well of a 12-well plate and the prepared reagent as a blank control (in triplicate wells) were transferred in 200 µL aliquots to four wells of a 96-well plate, resulting in a total of 12 wells in the 96-well plate. The absorbance of each well was measured once using a Sunrise microplate reader (30190079; Tecan, Männedorf, Switzerland) at a wavelength of 450 nm. Absorbance values were corrected by subtracting those of media-only blank wells, and the N/C group was set to 100% to normalize the relative cell viability for graphical and statistical analyses. Each condition was analyzed in triplicate (*n* = 3).

### 4.8. Colony Counting and AP Staining

Colonies were counted based on the following criteria: round shape with clear boundaries from feeder cells and a diameter of ≥50 µm. On day 7, colonies with diameters ≥ 80 µm were additionally distinguished and counted. Colony counts were performed in 12 independent fields (*n* = 12). AP staining was performed using the StemTAG™ Alkaline Phosphatase Staining Kit (CBA-300; Cell Biolabs, San Diego, CA, USA), according to the manufacturer’s protocol. The AP-stained cells were observed under a microscope (Nikon, Tokyo, Japan). The staining results were reproducible across three independent experiments, and representative images are shown.

### 4.9. Colony and pFeeder Isolation

pSSC colonies were initially isolated using a 200 µL pipette, ensuring that the pFeeders were not unintentionally detached during this process. Step 1 in Supplementary Figure S3A shows representative images of colonies isolated using pipette-based separation. The isolated colonies were then pre-plated on gelatin-coated 150 mm dishes for 1 h and 30 min, allowing feeder cells to adhere while maintaining the non-adherent colonies. Step 2 in Supplementary Figure S3A shows the colonies after the pre-plating step. Non-adherent colonies were collected and used for cell cycle analysis and RNA and protein extraction. The pFeeders were collected separately using 0.25% trypsin and pre-plated on 0.1% gelatin-coated plates for 1 h and 30 min. After washing three times with DPBS, the adherent cells were collected using 0.25% trypsin. The isolated pFeeders were used as controls to verify colony separation through RNA extraction and were also used for cell cycle analysis.

### 4.10. Cell Cycle Analysis

Colonies and pFeeders were isolated using the method described in Section 4.9. The colonies were dissociated into single cells by treatment with 0.05% trypsin at 37 °C for 5 min. The harvested cells were fixed in 70% ethanol at 4 °C for 3 min, washed, and resuspended in DPBS containing 50 μg/mL propidium iodide (PI; 287075; Sigma-Aldrich) and 0.1 mg/mL RNase A (EN0531; Thermo Fisher Scientific). The mixture was then incubated at RT for 15 min. The cell cycle of a minimum of 1 × 10^4^ cells was analyzed using a CytoFlex Flow Cytometry Analyzer (Beckman Coulter, Brea, CA, USA) with CytExpert 2.6 software (Beckman Coulter). All analyses were performed in triplicate (*n* = 3), and representative histograms are shown in Supplementary Figure S2. DNA content histograms obtained from PI staining were examined manually to distinguish different cell cycle phases: the major peak corresponding to diploid DNA content (2N) was designated as G0/G1, the intermediate region between G0/G1 and G2/M peaks was assigned as S phase, and the second peak corresponding to tetraploid DNA content (4N) was designated as G2/M.

### 4.11. Total RNA Extraction, RT-PCR and qPCR

Total RNA was extracted from the samples using an RNeasy Mini Kit (74104; Qiagen, Hilden, Germany), followed by DNase treatment. cDNA was synthesized from 2 μg of total RNA using a SuperScript III Reverse Transcriptase Kit (Invitrogen, Carlsbad, CA, USA) with oligo dT primers, according to the manufacturer’s instructions. PCR amplification was performed using an Applied Biosystems Veriti™ 96-Well Thermal Cycler (4452300; Thermo Fisher Scientific). For *B2M* and *WT1*, 30 cycles of 30 s at 95 °C, 20 s at 56 °C, and 30 s at 72 °C were performed. For *PGP9.5*, *THY-1*, *NANOG*, *PLZF*, *Gfra1*, *VASA*, and *CD9*, 35 cycles were performed under identical conditions. qPCR was performed using cDNA prepared from 2 μg of total RNA diluted five-fold with water. One microliter of diluted cDNA was used as a template, and 10 μL of AccuPower® 2X GreenStar™ qPCR MasterMix (K-6254; Bioneer, Daejeon, Republic of Korea) was used for qPCR using a Rotor-Gene Q (9001862; Qiagen). Cycling conditions were as follows: initial denaturation and polymerase activation at 94 °C for 10 min; followed by 40 cycles of 94 °C for 10 s, 56 °C for 10 s, 72 °C for 20 s; and final extension at 72 °C for 20 s. All cycle threshold (Ct) values were normalized to those of porcine *B2M*. Data are expressed as target gene expression relative to control gene expression. All qPCR experiments were performed in quadruplicate (*n* = 4). The primers used to detect the porcine transcripts are listed in Table 1.

### 4.12. Western Blotting

Colony cells were lysed in ice-cold RIPA buffer supplemented with cOmplete™ Protease Inhibitor Cocktail (11836170001; Roche, Basel, Switzerland). Total protein was quantified using a BCA Protein Assay Kit (23225; Pierce Biotechnology, Rockford, IL, USA). Each protein sample (30 µg) was loaded onto a 4–20% Mini-PROTEAN® TGX™ Precast Protein Gel. The proteins were then transferred to a polyvinylidene difluoride membrane and blocked by incubation in TBST buffer (20 mM Tris-HCl pH 7.5, 150 mM NaCl, and 0.1% Tween-20) with 5% BSA for 1 h at RT. The membranes were incubated overnight at 4 °C with primary antibodies diluted in TBST and 1% BSA (primary antibodies: PGP9.5 [7863-1004], Invitrogen; PLZF [PA5-29213], R&D Systems; GFRα-1 [sc-271546], Santa Cruz Biotechnology, Dallas, TX, USA; and β-actin [SC-47778], Santa Cruz Biotechnology). After washing the membranes three times with TBST, horseradish peroxidase (HRP)-linked anti-goat IgG (1:2000 dilution; sc-2354; Santa Cruz Biotechnology) to PLZF and HRP-linked anti-mouse IgG (1:2000 dilution; 7076P2; Cell Signaling, Danvers, MA, USA) to PGP9.5, GFRα-1, and β-actin, were added for 1 h 30 min at RT. Protein bands were visualized using a Bio-Rad ChemiDoc™ XRS+ imaging system and Image Lab™ Software version 6.1 (Bio-Rad, Hercules, CA, USA), with Pierce™ ECL Western Blotting Substrate (32106; Thermo Fisher Scientific) for β-actin, and SuperSignal™ West Femto Maximum Sensitivity Chemiluminescent Substrate (34096; Thermo Fisher Scientific) for PGP9.5, GFRA1, and PLZF. The band intensity of each target protein was measured using ImageJ software version 1.54g (National Institutes of Health, Bethesda, MD, USA). All band intensity values were normalized to those of β-actin. Data are expressed as target protein expression relative to that of the N/C control group, which was set to 1. Each analysis was repeated four times with similar results (*n* = 4).

### 4.13. Statistical Analysis

The R_a_ values obtained by AFM were analyzed using a sample size of *n* = 4. Colony counting was performed with a sample size of *n* = 12, WST-1 and cell cycle assays were conducted with *n* = 3, and qPCR and Western blotting analyses were conducted with *n* = 4. Statistical analyses were performed using GraphPad Prism version 8.0.2 (GraphPad Software, La Jolla, CA, USA). One-way analysis of variance was used to analyze the WST-1 assay, colony counting, qPCR, and Western blotting results, followed by Tukey’s multiple comparison test to assess the differences among groups. An unpaired *t*-test with Welch’s correction was used to analyze the colony number results. All data are expressed as mean ± SEM. Statistical significance is indicated by * *p* < 0.05, ** *p* < 0.01, and *** *p* < 0.001. The null hypothesis was rejected when the probability was *p* < 0.05.

## 5. Conclusions

In this study, we demonstrated that pECM derived from porcine foot tissue provides a supportive microenvironment for the in vitro culture of pSSCs. Compared to gelatin or N/C conditions, pECM promoted the formation of larger, well-defined colonies and supported AP-positive colony formation. Under pECM conditions, feeder cell overgrowth was suppressed, whereas pSSC proliferation was promoted. Furthermore, the expression of key stemness-related and germline-associated markers, including *PGP9.5*, *Thy-1*, *PLZF*, *Gfra1*, *NANOG*, and *VASA*, was enhanced, indicating that pSSCs were maintained in an undifferentiated state with active proliferation. Collectively, these findings highlight the potential of pECM as an effective coating material for SSC culture systems, particularly in pigs, in which feeder-free protocols have not yet been established.

## Figures and Tables

**Table 1:** Primers used for RT-PCR and qPCR.

Gene	Sequence	Tm (°C)	Product Size (bp)	GeneBank Accession
*B2M*	F 5′-TTCACACCGCTCCAGTAG-3′	55	166	NM_213978.1
	R 5′-CCAGATACATAGCAGTTCAG-3′	55		
*PGP9.5*	F 5′-GATGCCTTTTCCGGTGAACC-3′	59	173	NM_213763.2
	R 5′-GAACGGGGATAAAGCGAAGG-3′	59		
*Thy-1*	F 5’-GCTAACAGTCTTGCAGGTGG-3′	59	114	NM_001146129.1
	R 5′-ATGGGCAGGTTGGTGGTATT-3′	57		
*PLZF*	F 5′-GGCTCGGTATCTCAAGAACATC-3′	60	188	XM_021062870.1
	R 5′-ACTGCCCTATGGTCATCAAACT-3′	58		
*Gfra1*	F 5′-GCCACCAAGCGTTGTCTTTT-3′	57	175	XM_013983780.2
	R 5′-GAAGAGAGGGCCACATGTCC-3′	61		
*NANOG*	F 5′-ACCACTGGCCAAGGAATAGC-3′	59	111	NM_001129971.2
	R 5′-GCAGGTTTCCAGAAGCGTTC-3′	59		
*VASA*	F 5′-CCAAGTTGGCCAGTACTCAAAA-3′	58	214	NM_001291682.1
	R 5′-ACACTTCCCACAGCGAAAATC-3′	57		
*CD9*	F 5′-TTCATCTTCTGGCTCGCTGG-3′	59	164	NM_214006.1
	R 5′-AAGCCCACCACCATCATGAG-3′	59		
*WT1*	F 5′-CTGAAGACCCACACCAGGAC-3′	61	124	NM_001001264.1
	R 5′-GGTGCATGTTGTGATGACGG-3′	59		

## Data Availability

Data Availability Statement: The data supporting the findings of this study are available from the corresponding author upon reasonable request.

## Supplementary Materials

The following supporting information can be downloaded at: https://www.mdpi.com/article/10.3390/ijms26209937/s1.
