# Supplementary figures and images for: Effect of Extracellular Matrix Derived from Porcine Tissue on Stemness of Porcine Spermatogonial Stem Cells
Source: Int J Mol Sci. 2025 Oct 13;26(20):9937. doi: 10.3390/ijms26209937 (PMC12562855; doi:10.3390/ijms26209937)

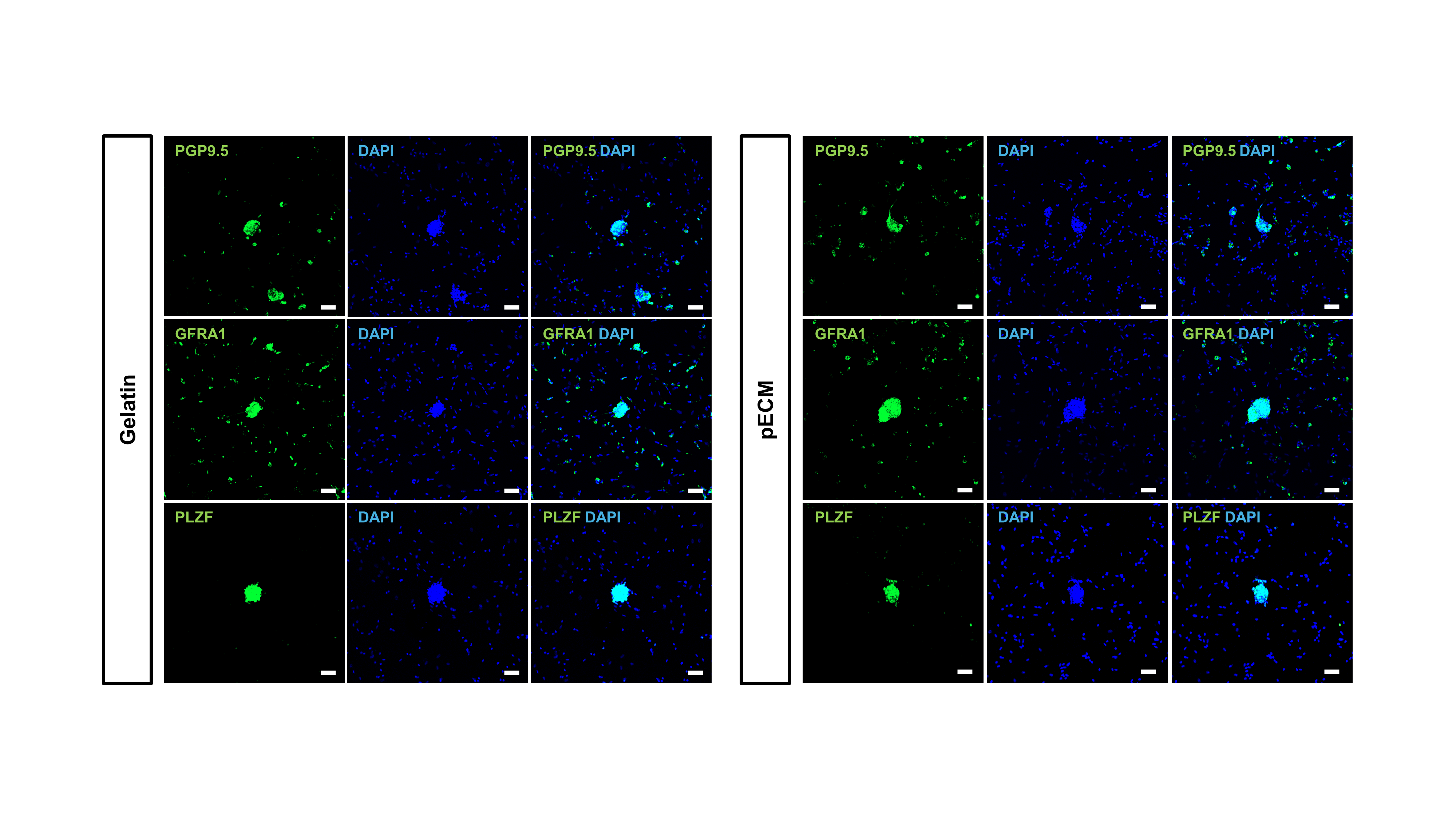

Supplement: Supplementary file 1 [file ijms-26-09937-s001.zip › SupplementaryFigure_S4.tif]

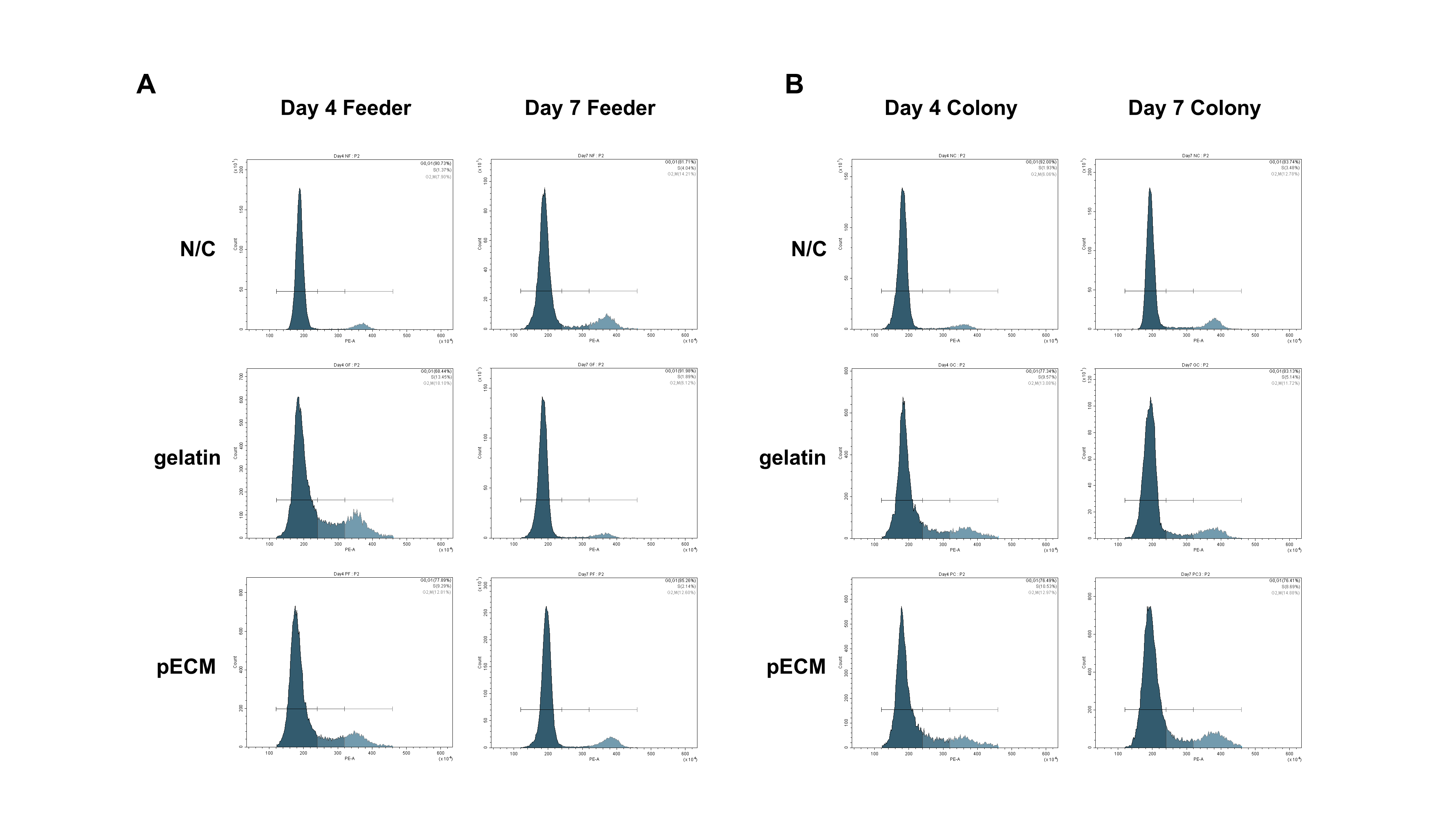

Supplement: Supplementary file 1 [file ijms-26-09937-s001.zip › SupplementaryFigure_S2.tif]

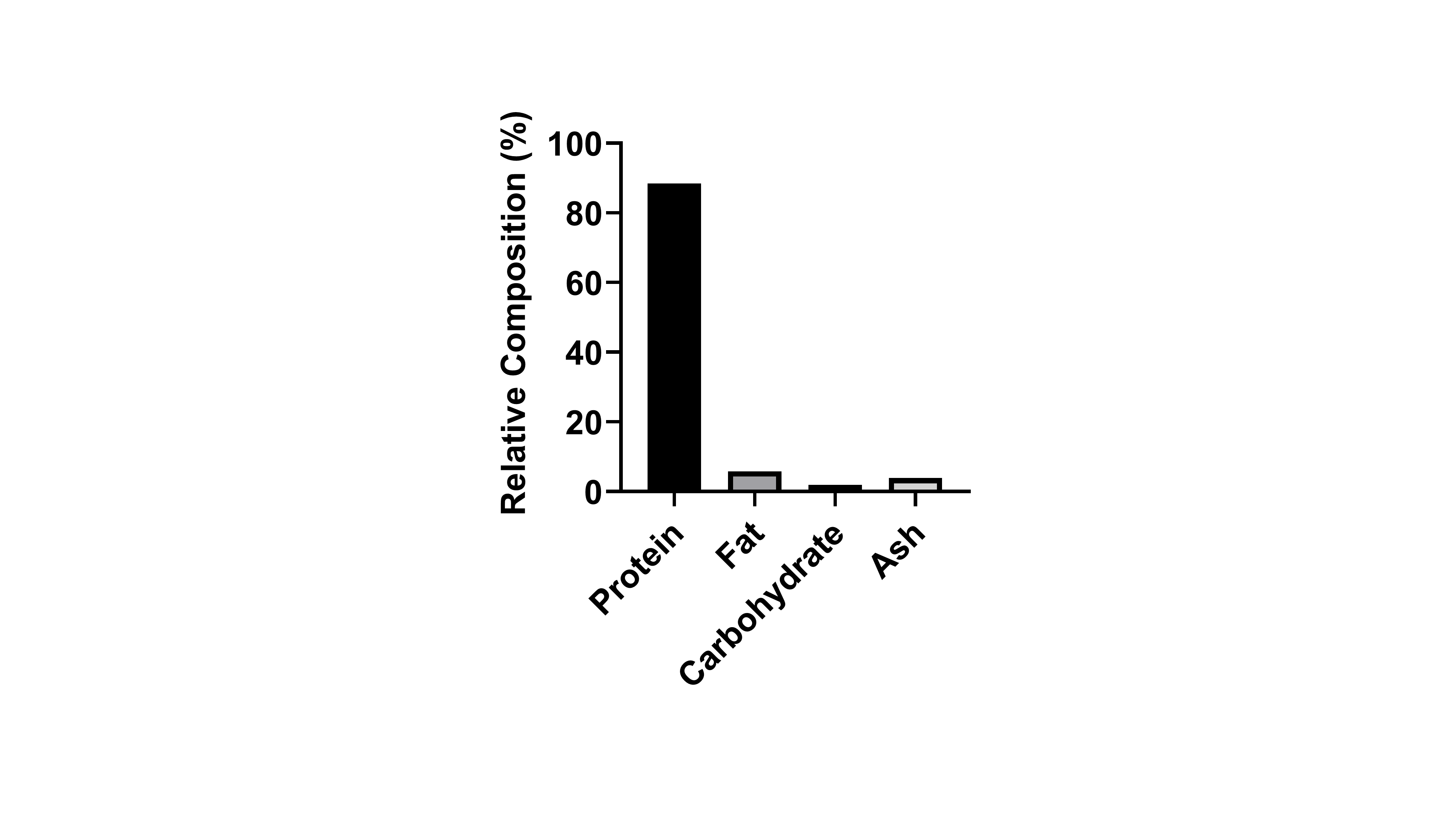

Supplement: Supplementary file 1 [file ijms-26-09937-s001.zip › SupplementaryFigure_S1.tif]

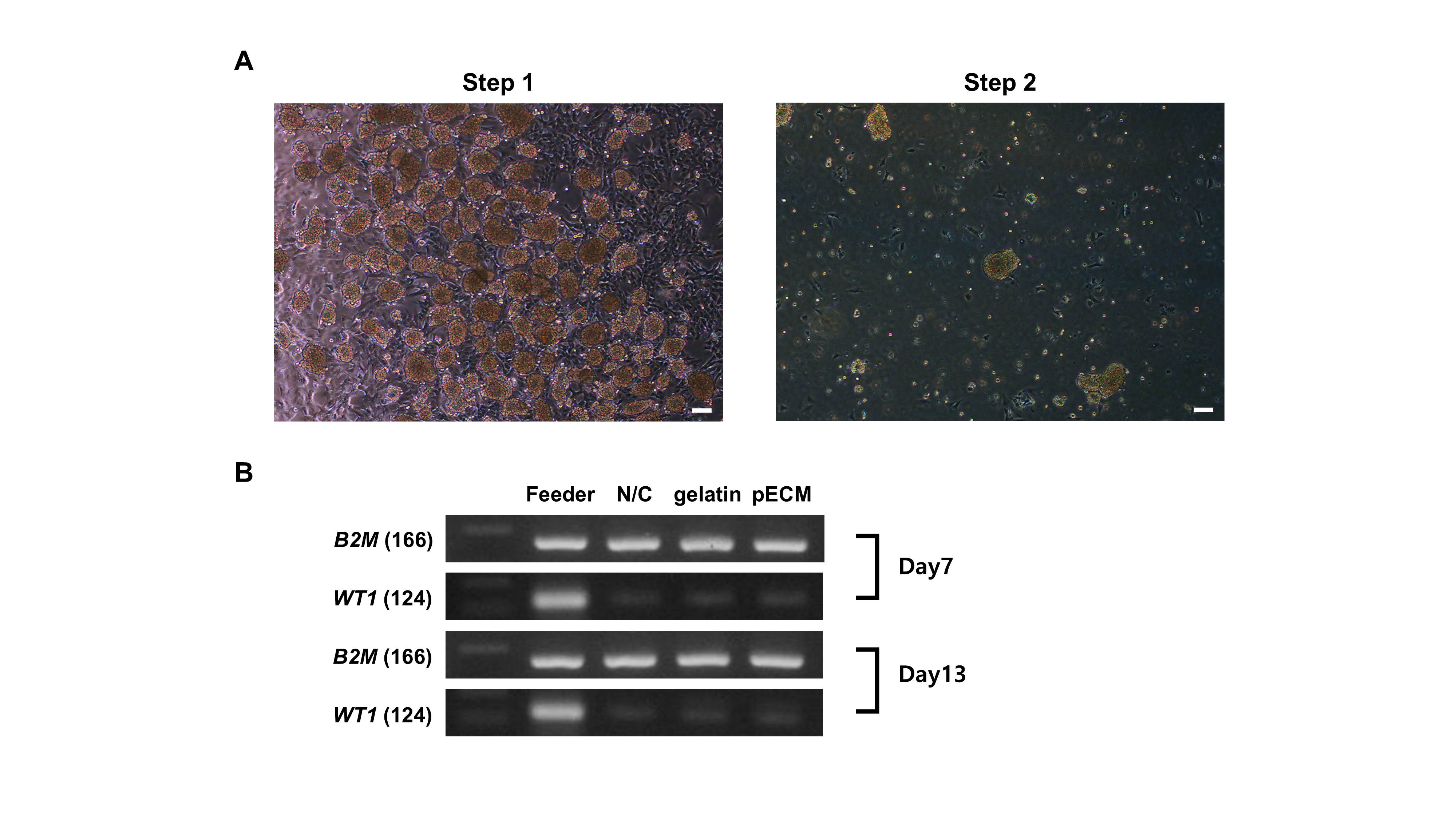

Supplement: Supplementary file 1 [file ijms-26-09937-s001.zip › SupplementaryFigure_S3.tif]
